# Impact of Endophytic Microorganisms on Plants, Environment and Humans

**DOI:** 10.1155/2014/250693

**Published:** 2014-01-22

**Authors:** Dhanya N. Nair, S. Padmavathy

**Affiliations:** Research Department of Botany, Nirmala College for Women, Coimbatore, Tamil Nadu 641018, India

## Abstract

Endophytes are microorganisms (bacteria or fungi or actinomycetes) that dwell within robust plant tissues by having a symbiotic association. They are ubiquitously associated with almost all plants studied till date. Some commonly found endophytes are those belonging to the genera *Enterobacter sp.*, *Colletotrichum sp.*, *Phomopsis sp.*, *Phyllosticta sp.*, *Cladosporium sp.,* and so forth. Endophytic population is greatly affected by climatic conditions and location where the host plant grows. They produce a wide range of compounds useful for plants for their growth, protection to environmental conditions, and sustainability, in favour of a good dwelling place within the hosts. They protect plants from herbivory by producing certain compounds which will prevent animals from further grazing on the same plant and sometimes act as biocontrol agents. A large amount of bioactive compounds produced by them not only are useful for plants but also are of economical importance to humans. They serve as antibiotics, drugs or medicines, or the compounds of high relevance in research or as compounds useful to food industry. They are also found to have some important role in nutrient cycling, biodegradation, and bioremediation. In this review, we have tried to comprehend different roles of endophytes in plants and their significance and impacts on man and environment.

## 1. Introduction

Endophytes are bacterial or fungal microorganisms that colonize healthy plant tissue intercellularly and/or intracellularly without causing any apparent symptoms of disease [1]. They are ubiquitous, colonize in all plants, and have been isolated from almost all plants examined till date. Their association can be obligate or facultative and causes no harm to the host plants. They exhibit complex interactions with their hosts which involves mutualism and antagonism [2–11]. Plants strictly limit the growth of endophytes, and these endophytes use many mechanisms to gradually adapt to their living environments [12]. In order to maintain stable symbiosis, endophytes produce several compounds that promote growth of plants and help them adapt better to the environment [13, 14].

Improvement of endophyte resources could bring us a variety of benefits, such as novel and effective bioactive compounds that cannot be synthesized by chemical reactions. For this, there should be a better understanding about endophytes, their significance and roles. Understanding the biology of plants and their microbial ecology becomes important. As evidenced by more number of publications on endophytes in recent years, many studies have been performed for evaluating their colonization pattern of vegetative tissues as well as their effects on plant growth. These publications indirectly suggest their importance to the hosts and to the environment. This review aims to provide an overview about endophytes, their role and importance in plants and subsequently to the environment and human beings with reference to recent developments in endophytic research.

## 2. Isolation and Identification

Endophytic organisms have been isolated from different parts of plant. They were isolated from scale primordia, meristem and resin ducts [15, 16], leaf segments with midrib and roots [17] and from stem, bark, leaf blade, petiole [18], and buds [19]. Sequence-based approach was used for investigating the transmission of diverse fungal endophytes in seed and needles of *Pinus monticola*, western white pine [20]. They isolated 2003 fungal endophytes from 750 surface-sterilized needles. In contrast, only 16 endophytic isolates were obtained from 800 surface-sterilized seeds.

There are endophytic bacteria, fungi, and/or actinomycetes whose isolation from the plant tissues has been a challenge since the studies on endophytes started. Several researchers have reviewed extensively different methods of the isolation of bacterial endophytes [21, 22]. Endophytes are isolated by initial surface sterilization followed by culturing from ground tissue extract [23] or by direct culturing of plant tissues [18] on media suitable for bacteria or fungi or actinomycetes. The impact of different culture media on isolation of endophytic fungal flora from root and fruits of *Azadirachta indica* A. Juss was studied by Verma et al. [24]. According to them, mycological agar (MCA) medium yielded the highest number of isolates, with the greatest species richness.

An obligatory endophyte, *Enterobacter cloacae, *was found to be associated with the pollen of several Mediterranean pines [25]. Most fungal endophytes isolated from plants and algae are members of the Ascomycota or their anamorphs, with only a few reports of basidiomycetous endophytes, these often being orchid mycorrhizas [26]. Basidiomycetous morphotypes were isolated from healthy leaves, rachis, and petioles of the oil palm *Elaeis guineensis *in a Thai plantation which were further characterized by molecular analysis using ribosomal DNA sequences. For the first time ever, the microorganism species *Acremonium terricola*, *Monodictys castaneae*, *Penicillium glandicola*, *Phoma tropica* and *Tetraploa aristata* were reported as endophytic fungi [27]. Some of the common and more frequently isolated endophytic fungi from different plants are given in Table 1.

Endophytic fungi have been classified into two broad groups discriminated based on phylogeny and life history traits as clavicipitaceous (C) which infect some grasses and the nonclavicipitaceous endophytes (NC-endophytes), which can be recovered from asymptomatic tissues of nonvascular plants, ferns and allies, conifers, and angiosperms [28]. NC-endophytes represent three distinct functional classes based on host colonization and transmission, *in planta *biodiversity and fitness benefits conferred to hosts while the C group has just one class.

Conventionally, identification of endophytes was done based on morphological characteristics for bacteria, fungi, and actinomycetes and with the help of biochemical tests for bacteria and actinomycetes. With the development of molecular biology, ribosomal DNA Internal Transcribed Spacer (ITS) sequence analysis is widely used for the identification of microorganisms. Ribosomal DNA (rDNA) ITS was proved to be a valuable source of evidence to resolve phylogenetic relationships at lower levels, such as among genera or species [29]. It was also reported that ITS sequences analysis was especially effective in nonsporulating fungi identification which reduced the impact of biased judgement [30] and the Large Subunit (LSU) and ITS data are powerful tools to resolve the taxonomy of basidiomycetous endophytes [26]. *Pleurostoma, Chaetomium, Coniochaeta (Lecythophora), Daldinia, Xylaria, Hypoxylon, Nodulisporium, Cazia, *and* Phellinus* isolated as endophytes from *Huperzia serrata* were confirmed for the first time by rDNA ITS analysis [31].

## 3. Effect of Climate on Endophytic Population

Endophytic population varies from plants to plants and from species to species. Within the same species it not only varies from region to region but also differs with change in climatic conditions of the same region. Temporal changes in relative frequency of total endophytic fungi were studied by Chareprasert et al. [32]. They found that matured leaves of teak (*Tectona grandis* L.) and rain tree (*Samanea saman* Merr.) had greater number of genera and species, with higher colonization frequency, than those in the young leaves and their occurrence in leaves increased during rainy season. The endophytic population and frequency tended to differ among sampling dates for all the organs studied, namely, young leaves, petiole, and twigs of *Gingko biloba* L. [33]. They proved that the occurrence of *Phyllosticta* sp. in both leaves and petioles was first detected in August and peaked in October with none in the month of May. *Phomopsis* sp. was detected in twigs throughout the growing season. These results suggest that the distribution of the two dominant endophytic fungi was organ-specific and differed within seasons.

## 4. Endophytes and Molecular Studies 

With recent advances and developments in biotechnology, more studies at the molecular level are done with endophytes, which include metagenomic studies, use of molecular markers, molecular cloning, and genetic expression studies. Denaturing gradient gel electrophoresis (DGGE) profiles of 16S rRNA gene fragments amplified from total plant DNA were used to detect some nonculturable endophytic bacteria by comparing the profile with the bands obtained from the culturable endophytes from Citrus plant [34]. Bacterial automated ribosomal intergenic spacer analysis (B-ARISA) and Pyrosequencing was used to examine bacterial endophyte community of potato (*Solanum tuberosum*) cultivar [35]. B-ARISA profiles revealed a significant difference in the endophytic community between cultivars and canonical correspondence analysis showed a significant correlation between the community structure and plant biomass. Pyrosequencing was used to determine the bacterial operational taxonomic units (OTUs) richness. Metagenomic approach is another method used to find the microorganisms from different environments which cannot be cultured easily. This approach was used to find the 1-aminocyclopropane-1-carboxylate deaminase gene (*acdS*) operon from an uncultured endophytic microorganism colonizing *Solanum tuberosum* L. [36]. The authors in [36] concluded that metagenomic analysis can complement PCR-based analysis and yield information on whole gene operons.

Little variation within the endophytic population diversity in *Festuca eskia* was found, regardless of provenance altitude and site and/or endophyte infection frequency using Sequence Tagged Sites (STS) and Simple Sequence Repeats (SSR) markers [37]. SSR marker was also used to study the genetic variation among two isolated endophytes *Neotyphodium sibiricum *and *N. gansuense* from the host plant *Achnatherum sibiricum* [38]. Significant linkage disequilibrium of fungal SSR loci suggested that both fungal species primarily propagate by clonal growth through plant seeds, whereas variation in genetic diversity and the presence of hybrids in both endophytic species revealed that, although clonal propagation was prevalent, occasional recombination might also occur. Based on molecular cloning and genetic expression of analysis of geranylgeranyl diphosphate (GGPP) synthase, it was proposed that *ltmG, ltmM,* and *ltmK* are members of a set of genes required for lolitrem (a potent tremorgen to mammals) biosynthesis in endophytes *Epichloe festucae* and *Neotyphodium lolii* of the perennial ryegrass [39]*.*

Molecular studies in endophytes have gone to the extent that complete genome of *Enterobacter* sp. 638, an endophytic plant growth promoting gamma-proteobacterium, that was isolated from the stem of poplar, a potentially important biofuel feed stock plant, was sequenced [40]. Sequencing revealed that it has 4,518,712 bp chromosome and a 157,749 bp plasmid (pENT638-1). Different sets of genes specific to the plant niche adaptation of this bacterium were identified by genome annotation and comparative genomics. This includes genes that code for putative proteins involved in survival in the rhizosphere (to cope with oxidative stress or uptake of nutrients released by plant roots), root adhesion (pili, adhesin, hemagglutinin, and cellulose biosynthesis), colonization/establishment inside the plant (chemiotaxis, flagella, and cellobiose phosphorylase), plant protection against fungal and bacterial infections (siderophore production and synthesis of the antimicrobial compounds 4-hydroxybenzoate and 2-phenylethanol), and overall improved poplar growth and development (through production of the phytohormones indole acetic acid, acetoin, and 2,3-butanediol).

## 5. Roles and Applications of Endophytes

### 5.1. Phytostimulation

Plants require 16 essential elements like C, H, N, O, and P and 11 more. These essential elements are available to plants for their growth and development in chemical form, which they obtain from atmosphere, soil, water, and organic matter. Endophytes also play an important role in the uptake of these nutrients. They elicit different modes of action in tall fescue adaptation to P deficiency [41] and induce increased uptake of N [42]. Endophytic bacteria produce a wide range of phytohormones, such as auxins, cytokinins, and gibberellic acids. *Burkholderia vietnamiensis*, a diazotrophic endophytic bacterium isolated from wild cottonwood (*Populus trichocarpa*), produced indole acetic acid (IAA), which promotes the growth of the plant [43]. This was confirmed by comparison between uninoculated control and plants inoculated with *B. vietnamiensis* on nitrogen free media, in which inoculated plants gained more dry weight and more nitrogen content. A new strain of fungus *Cladosporium sphaerospermum* isolated from the roots of *Glycine max* (L) Merr. showed the presence of higher amounts of bioactive GA3, GA4, and GA7, which induced maximum plant growth in both rice and soybean varieties [44].

### 5.2. Pigment Production

An orange pigment identified as quercetin glycoside was isolated from an endophytic fungus belonging to *Penicillium sp.* [45]. This was the first report on quercetin glycoside produced by endophytic fungus. Endophytic fungus strain named SX01, later identified as *Penicillium purpurogenum*, from the twigs of *Ginkgo biloba* L, was able to produce abundant soluble red pigments which could be used as natural food colorant [46]. A pigment isolated from the endophytic fungus *Monodictys castaneae* was found to inhibit few human pathogenic bacteria *Staphylococcus aureus*, *Klebsiella pneumonia, Salmonella typhi, *and *Vibrio cholerae *and was proved to be more active than streptomycin [47].

### 5.3. Enzyme Production

Many commercially important enzymes are produced by several soil micro-organisms. The hunt for other potential sources had led to the discovery of a few vital enzymes being produced by endophytes. Endophytic fungi like *Acremonium terricola*, *Aspergillus japonicas*, *Cladosporium cladosporioides*, *Cladosporium sphaerospermum*, *Fusarium lateritium*, *Monodictys castaneae*, *Nigrospora sphaerica*, *Penicillium aurantiogriseum, Penicillium glandicola, Pestalotiopsis guepinii, Phoma tropica, Phomopsis archeri, Tetraploa aristata,* and *Xylaria sp.* and many other unidentified species in *Opuntia ficus-indica* Mill. have indicated their promising potential for deployment in biotechnological processes involving production of pectinases, cellulases, xylanases, and proteases [27]. An endophyte, *Acremonium zeae*, isolated from maize produced the enzyme hemicellulase extracellularly [48]. This hydrolytic enzyme from *A. zeae* may be suitable for application in the bioconversion of lignocellulosic biomass into fermentable sugars.

### 5.4. Antimicrobial Activity

Most of the endophytes isolated from plants are known for their antimicrobial activity. They help in controlling microbial pathogens in plants and/or animals. Endophytes isolated from medicinal plants showed bioactivity for broad spectrum of pathogenic microorganisms [49–51]. A total of 37 endophytes were isolated all together from *Tectona grandis* L. and *Samanea saman* Merr. of which 18 could produce inhibitory substances effective against *Bacillus subtilis, Staphylococcus aureus,* and *Escherichia coli* and 3 isolates inhibited growth of *Candida albicans in vitro* [32]. Kumar et al. [52] assayed the bioactivity of the endophytic microorganisms like *Dothideomycetes sp*., *Alternaria tenuissima*, *Thielavia subthermophila*, *Alternaria sp.*, *Nigrospora oryzae*, *Colletotrichum truncatum*, and *Chaetomium sp*., isolated from the medicinal plant, *Tylophora indica*, against *Sclerotinia sclerotiorum* and *Fusarium oxysporum *which were found to inhibit their growth.

### 5.5. Source of Bioactives and Novel Compounds

Endophytes are capable of synthesizing bioactive compounds that are used by plants for defence against pathogens and some of these compounds have proven to be useful for novel drug discovery. Recent studies have reported hundreds of natural products including alkaloids, terpenoids, flavonoids, and steroids, from endophytes. Most of the bioactive compounds isolated from endophytes are known to have functions of antibiotics, immunosuppressants, anticancer agents, biological control agents, and so forth [53].

**Figure 1:**
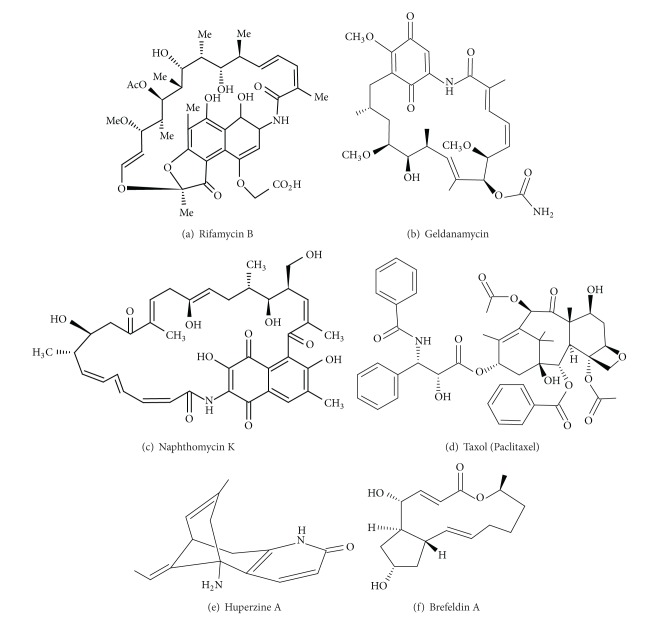
Chemical structures of some bioactive compounds produced by endophytic microorganisms.

Maytansinoids, like rifamycin (Figure 1(a)) and geldanamycin (Figure 1(b)), which structurally belong to the ansamycin family of polyketide macrolactams are products of three closely related plant families (Celastraceae, Rhamnaceae, and Euphorbiaceae), mosses, and certain bacteria such as *Actinosynnema pretiosum*. It was hypothesized that microbes in the rhizosphere might be involved in the biosynthesis of plant maytansinoids [54]. Several endophytic actinomycetes were isolated from *Trewia nudiflora*, of which *Streptomyces* sp. 5B and *Streptomyces* sp. M27m3 were proved to have the potential of producing ansamycins [42]. One novel chlorine-containing ansamycin, namely, naphthomycin K (Figure 1(c)), which was isolated from the endophytic strain *Streptomyces* sp. CS of the maytansinoids producer medicinal plant *Maytenus hookeri*, showed evident cytotoxic activity against P388 and A-549 cell lines, but no inhibitory activities against *Staphylococcus aureus* and *Mycobacterium tuberculosis* [55].

Siderophores are biologically active compound with function of chelating iron ions in living organisms. They have found extensive applications in the field of agriculture and medicine. They are also a component of virulence of microorganisms infecting man, animals, and plants [56]. Five different strains of *Phialocephala fortinii*, a dark septate fungal, were studied and all of them excreted three siderophores namely, ferricrocin, ferrirubin and ferrichrome C, whose production was dependent on pH and iron(III) concentration of the culture medium [57]. *P. fortinii* can thus be used for large scale production of these siderophores.

Taxol (Figure 1(d)) is a drug used to cure breast cancer, ovarian cancer, and lung cancer. An endophytic microorganism *Metarhizium anisopliae*, isolated from *Taxus chinensis*, was found to produce taxol in abundance *in vitro *[58]. *Colletotrichum gloeosporioides* isolated from the leaves of a medicinal plant, *Justicia gendarussa,* also produces taxol [59].

Huperzine A (HupA) (Figure 1(e)), a lycopodium alkaloid was isolated originally from* Huperzia serrata*. It has attracted intense attention since its marked role as cholinesterase inhibitor was discovered [60, 61]. Over 120 endophytic fungi were recovered from *H. serrata* and when screened for Hup-A, nine of them produced it [62]. From the all screened fungi, *Shiraia sp.* was found to be the most significant producer of HupA.

Two new benzopyranones, diaportheone A and B, were obtained via bioassay-guided isolation of the secondary metabolites from the endophytic fungus *Diaporthe sp*. P133 isolated from *Pandanus maryllifolius* leaves. Compounds diaportheone A and B inhibited the growth of the virulent strain of *Mycobacterium tuberculosis* H37Rv with minimum inhibitory concentrations of 100.9 and 3.5 *μ*M, respectively [63].

Brefeldin A (Figure 1(f)) is a macrocyclic lactone synthesized from palmitate by a variety of fungi, which inhibits the protein secretion in the cells [64]. The membrane protein of the cell is retained in endoplastic reticulum from where it is not transported to Golgi apparatus and also results in retrotransport of the proteins in golgi complex, which have been secreted before the treatment of cells with it, to endoplasmic reticulum. It was initially known as an antiviral drug but lately was used to study protein synthesis and secretion in cells. Brefeldin A was isolated from active endophytic strain I(R)9-2, *Cladosporium sp*. from *Quercus variabilis* [65].

Nine new biologically active secondary metabolites were isolated from endophytic fungus *Alternaria alternata* residing in *Maytenus hookeri *and were characterized by NMR as (i) alternariol, (ii) alternariol monomethyl ether, (iii) 5-epialtenuene, (iv) altenuene, (v) uridine, (vi) adenosine, (vii) ACTG toxin-E, (viii) ergosta-4,6,8,22-tetraen-3-one, and (ix) ergosta-7,24(28)-dien-3-ol from the ethyl acetate-methanol-acetic acid extract of the solid-state fermentations of this fungus [66].

### 5.6. Reciprocal Interactions between Above- and Belowground Communities

The microbial community responses in soils conditioned by plants of the annual grass *Lolium multiflorum* with contrasting levels of infection with the endophyte *Neotyphodium occultans* were explored [67]. Soil conditioning by highly infected plants affected soil catabolic profiles and tended to increase soil fungal activity. A shift in bacterial community structures was detected while no changes were observed for fungi. Soil responses became evident even without changes in host plant biomass or soil organic carbon or total nitrogen content, suggesting that the endophyte modified host rhizo depositions during the conditioning phase. A few researchers have reported changes in the rhizosphere chemistry and enzymatics activity mediated by endophyte presence in perennial host grasses [68, 69].

### 5.7. Biocontrol Agents

Endophytic microorganisms are regarded as an effective biocontrol agent, alternative to chemical control. Endophytic fungi have been described to play an important role in controlling insect herbivory not only in grasses [5] but also in conifers [70]. An endophytic fungi *Beauveria bassiana *known as an entomopathogen was found to control the borer insects in coffee seedlings [70] and sorghum [71]. The fungal pathogen *Botrytis cinerea* causes severe rotting on tomato fruits during storage and shelf life. The endophytic bacteria *Bacillus subtilis*, isolated from *Speranskia tuberculata* (Bge.) Baill, was found to be strongly antagonistic to the pathogen * B. cinerea* in* in vitro *studies[72]. A new strain of *Burkholderia pyrrocinia* JK-SH007 and *B. cepacia,* were identified as potential biocontrol agent against poplar canker [73].

Not only naturally occurring endophytes are used as biocontrol agents but also they are genetically engineered to express antipest proteins like lectins. Initial attempts were made to introduce heterologous gene into an endophytic microorganism for insect control [74, 75]. With the advent of time, many scientists have worked on this aspect, which has been one of the important studies in endophytes recently. Fungal endophyte of *Chaetomium globosum *YY-11 with antifungi activities, isolated from rape seedlings, and bacterial endophytes of *Enterobacter *sp. and *Bacillus subtilis* isolated from rice seedlings were used to express *Pinellia ternate *agglutinin (*PtA*) gene [76]. These recombinant endophytes expressing *PtA* gene were found to effectively control the population of sap sucking pests in several crop seedlings. Similarly, in a different study, recombinant endophytic bacteria *Enterobacter cloacae* expressing *PtA* gene proved to be a bioinsecticide against white backed planthopper, *Sogatella furcifera *[77]. Use of recombinant endophytes as biocontrol agents expressing different antipest proteins becomes a promising technique for control of plant pests as these endophytes can easily colonize within different crop plants successfully.

### 5.8. Nutrient Cycling

Nutrient cycling is a very important process that happens continuously to balance the existing nutrients existing and make it available for every component of the ecosystem. Biodegradation of the dead biomasses becomes one major step in it to bring back the utilized nutrients back to the ecosystem which in turn again becomes available to the organisms. This becomes a cyclic chain process. A lot of saprophytic organisms play a major role in it. Few studies have shown that endophytes have important role in biodegradation of the litter of its host plants [78–85]. During biodegradation of the litter, the endophytic microbes colonize initially within the plants [86] and facilitate the saprophytic microbes to act on through antagonistic interaction, and thus increasing the litter decomposition [87, 88]. In another study, it was demonstrated that all endophytes had the ability to decompose organic components, including lignin, cellulose, and hemicellulose however the preferences of various groups of endophytes with respect to organic compounds differed [89].

### 5.9. Bioremediation/Biodegradation

Endophytes have a powerful ability to breakdown complex compounds. Bioremediation is a method of removal of pollutants and wastes from the environment by the use of micro-organisms. It relies on the biological processes in microbes to breakdown these wastes. This is made possible due to the great microbial diversity. A group of researchers studied the role of endophytes in bioremediation in *Nicotiana tabaccum* plants [90]. Inoculation of *Nicotiana tabaccum* with endophytes resulted in improved biomass production under conditions of Cadmium (Cd) stress, and the total plant Cd concentration was higher compared to noninoculated plants. These results demonstrated the beneficial effects of seed endophytes on metal toxicity and accumulation.

To explore the endophytic diversity for the breakdown of plastic, several dozen endophytic fungi were screened for their ability to degrade the synthetic polymer polyester polyurethane (PUR) [91]. Though several organisms demonstrated the ability to efficiently degrade PUR in both solid and liquid suspensions, robust activity was observed among several isolates in the genus *Pestalotiopsis*. Two *Pestalotiopsis microspora *isolates were uniquely able to grow on PUR as the sole carbon source under both aerobic and anaerobic conditions. Molecular characterization of this activity suggested that an enzyme serine hydrolase is responsible for degradation of PUR [91].

### 5.10. Production of Volatile Organic Compounds and Their Benefits

*Hypoxylon sp*. which is an endophytic fungus isolated from *Persea indica* produced an impressive spectrum of volatile organic compounds (VOCs), most notably 1,8-cineole, 1-methyl-1,4-cyclohexadiene, and tentatively identified alpha-methylene-alpha-fenchocamphorone, among many others, most of which are unidentified. It displayed maximal VOC antimicrobial activity against *Botrytis cinerea*, *Phytophthora cinnamomi*, *Cercospora beticola*, and *Sclerotinia sclerotiorum* suggesting that the VOCs may play some role in the biology of the fungus and its survival in its host plant [92]. They unequivocally demonstrated that 1,8-cineole (a monoterpene) is produced in addition by this *Hypoxylon sp.*, which represents a novel and important source of this compound. This monoterpene is an octane derivative and has potential use as a fuel additive as do the other VOCs of this organism. This study thus shows that fungal sourcing of this compound and other VOCs as produced by *Hypoxylon sp*. greatly expands their potential applications in medicine, industry, and energy production.

An unusual *Phomopsis sp*. was isolated as endophyte of *Odontoglossum sp. *(Orchidaceae), produced a unique mixture of volatile organic compounds (VOCs) including sabinene (a monoterpene with a peppery odor), 1-butanol, 3-methyl; benzeneethanol; 1-propanol, 2-methyl, and 2-propanone [93]. The gases of *Phomopsis sp*. possess antifungal properties and an artificial mixture of the VOCs mimicked the antibiotic effects of this organism with the greatest bioactivity against a wide range of plant pathogenic test fungi including *Pythium*, *Phytophthora*, *Sclerotinia*, *Rhizoctonia*, *Fusarium*, *Botrytis*, *Verticillium*, and *Colletotrichum*. As with many VOC-producing endophytes, this *Phomopsis sp*. did survive and grow in the presence of the inhibitory gases of *Muscodor albus*, an endophytic fungus. The authors in [93] had hypothesized that there was a possible involvement of VOC production by the fungus and its role in the biology/ecology of the fungus/plant/environmental relationship.

### 5.11. Endophytes with Multiple Roles

Many endophytes are known to have wide range of activity within hosts. Endophytic microbes were found to have herbicidal activity along with antimicrobial activity [94]. *Bacillus sp*. SLS18, known as a plant growth-promoting endophyte, was investigated for its role in the biomass production and manganese and cadmium uptake by *Sorghum bicolor* L., *Phytolacca acinosa *Roxb., and *Solanum nigrum* L. [95]. It displayed multiple heavy metals and antibiotics resistances. The strain also exhibited the capacity of producing indole-3-acetic acid, siderophores, and 1-aminocyclopropane-1-carboxylic acid deaminase.

### 5.12. Endophytes in Tissue Culture

Endophytes are largely useful to the host plants in many ways as discussed in earlier part of this review. But when it comes to plant tissue culture it is usually considered as a contaminant. The ultimate aim of tissue culture is to develop axenic plants. Though we surface sterilize the explants to be used for tissue culture, after few days, bacteria or fungi or actinomycetes or all of them start growing from tissue or the cultured explant. These contaminants are nothing but endophytic microbes resulting in complete loss of time, media, and explants, which sometimes may be of some rare and endangered species which needs to be conserved by tissue culture techniques.

Endophyte species composition and plant genotype together with tissue culture conditions are the key factors for gaining plant tissue cultures with high regeneration capacity. Interaction between the endophytes and specific secondary compounds may be an important factor for browning and cell death in the Scots pine calli [19]. These researchers examined the green, light brown, and dark brown calli by TEM with respect to presence of microbial cells in the tissues. The microbial cells were encountered more frequently in cells of the brown tissues than in the green, well growing tissues, which suggested that endophytes could either be involved with browning by inducing the senescence and release of tannins in the tissue or that the endophytes take over the otherwise senescing callus tissue.

Plant tissue culture, which commonly utilizes the meristems, which are considered to be sterile part of any plant, has however given numerous references to microbial existence in these tissues [96–98]. Elimination of these endophytes thus becomes a prime objective to develop axenic plants during tissue culture. Many protocols have been developed by many researchers to overcome this problem.

## 6. Conclusions 

Recent years have seen great deal of interest among researchers in the studies on endophytic microorganisms, thanks to easier methods of isolation and identifications and current tools of molecular biology. Many bioactive compounds beneficial to pharmaceuticals, environment, agriculture, and industries are produced by endophytes. Due to their great importance to plants/human beings/environment, scientists have already started exploiting them very much for newer compounds and newer roles to the environment and human. It becomes sensible to review on past achievements in the field of endophytic research, opening up broader opportunities for the scientific community.

Endophytes can be either bacteria or fungi or actinomycetes. In this review, we have come to a conclusion that it is mostly the actinomycetes which are involved in production of pharmaceutically important compounds within the plants. Generally, the fungi are involved in the role of phytoremediation, biodegradation, and nutrient cycling and thus reduce the debris load on the environment in a better way. By and large, it is the bacterial community of endophytes which helps the plants in their better growth by producing different growth hormones. Application of different innovative biotechnological tools will help in strengthening the understanding of plant-endophyte interactions, producing new bioactive compounds, perk up the growth in plants, and improve biocontrol activity, reducing the debris and other wastes which are otherwise harmful to the ecosystem. Considering all these, definitely endophytes have proved to be a boon and have left good impact on plants, environment, and also human beings in several possible ways.

## Figures and Tables

**Table 1:** Fungi those are commonly isolated as endophytes from different plants.

Endophytes	Plant species	Citation
*Phomopsis sp *	*Neolitsea sericea *	[18]
	*Pasania edulis *	[17]
	*Ginkgo biloba *L*. *	[33]
	*Tectona grandis and Samanea saman *Merr.	[32]
	*Taxus chinensis *	[58]

*Cladosporium sp. *	*Opuntia ficus indica *	[27]
	*Cinnamomum camphora *	[89]
*C. herbarum *	*Lycopersicum esculentum *Mill*. *	[99]
	*Triticum aestivum *	[100]

*Colletotrichum sp. *	*Triticum aestivum *	[100]
	*Citrus plants *	[37]
	*Cinnamomum camphora *	[89]
	*Pasania edulis *	[17]
	*Ginkgo biloba *L*. *	[33]
	*Tectona grandis and Samanea saman *Merr*. *	[32]
	*Huperzia serrata *	[62]
	*Cinnamomum camphora *	[89]
*C. gloeosporiodes *	*Lycopersicum esculentum *Mill.	[99]

*Phyllosticta sp *	*Citrus sp. *	[37]
	*Pasania edulis *	[17]
	*Coffea arabica *	[101]
	*Quercus variabilis *	[65]
	*Centella asiatica *	[102]
	*Panax quinquefolium *	[103]
	*Ginkgo biloba *L.	[33]

*Penicillium sp. *	*Lycopersicum esculentum *Mill.	[99]
	*Huperzia serrata *	[62]

*Acremonium sp. *	*Taxus chinensis *	[58]
	*Huperzia serrata *	[37]
